# An Ultrasonic Multi-Beam Concentration Meter with a Neuro-Fuzzy Algorithm for Water Treatment Plants

**DOI:** 10.3390/s151026961

**Published:** 2015-10-23

**Authors:** Ho-Hyun Lee, Sang-Bok Jang, Gang-Wook Shin, Sung-Taek Hong, Dae-Jong Lee, Myung Geun Chun

**Affiliations:** 1 School of of Electrical Engineering and Computer Science, Chungbuk National University, Cheongju 28644, Korea; E-Mails: Lhh@kwater.or.kr (H.-H.L.); bigbell@cbnu.ac.kr (D.-J.J.); 2 K-Water Research Institute, Korea Water Resources Corporation, Daejeon 34045, Korea; E-Mails: jsbok0502@kwater.or.kr (S.-B.J.); gwshin@kwater.or.kr (G.-W.S.); sthong@kwater.or.kr (S.-T.H.)

**Keywords:** ultrasonic concentration meter, neuro-fuzzy model, water treatment plants

## Abstract

Ultrasonic concentration meters have widely been used at water purification, sewage treatment and waste water treatment plants to sort and transfer high concentration sludges and to control the amount of chemical dosage. When an unusual substance is contained in the sludge, however, the attenuation of ultrasonic waves could be increased or not be transmitted to the receiver. In this case, the value measured by a concentration meter is higher than the actual density value or vibration. As well, it is difficult to automate the residuals treatment process according to the various problems such as sludge attachment or sensor failure. An ultrasonic multi-beam concentration sensor was considered to solve these problems, but an abnormal concentration value of a specific ultrasonic beam degrades the accuracy of the entire measurement in case of using a conventional arithmetic mean for all measurement values, so this paper proposes a method to improve the accuracy of the sludge concentration determination by choosing reliable sensor values and applying a neuro-fuzzy learning algorithm. The newly developed meter is proven to render useful results from a variety of experiments on a real water treatment plant.

## 1. Introduction

The sludges discharged from water, sewage and wastewater treatment plants are buried, dumped into the ocean or incinerated, and for this reason, urgent action is required to effectively reduce sludge emissions according to pollution regulations [1,2,3]. In general, the sludge treatment process involves precipitation from the sedimentation basin of the water treatment processes and it then goes through adjustment, concentration, dehydration and disposal [4]. Sludge is usually mixed with colloid material and hydroxide to create a coagulant which is then submerged and allows for the solid-liquid part to be separated from the agglomerated material. The main source of sludge from the water treatment plant stems from submerged and back-washed water and it is very important to know the amount of solids in the sludge through a density meter. Therefore, it is necessary to accurately measure the sludge concentration to optimize the polymer dosage rate and stabilize the water quality to be discharged to rivers [5,6].

**Figure 1:**
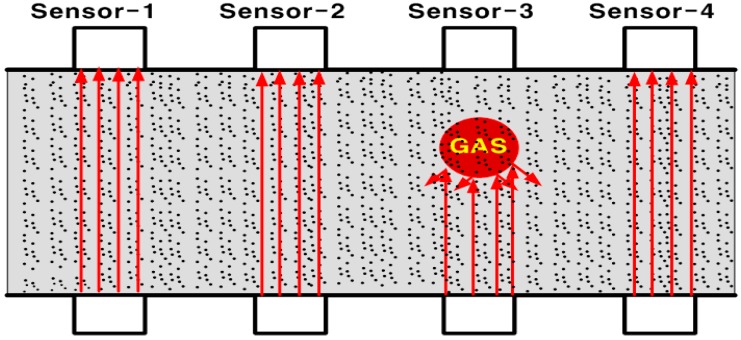
Example of sludge measurement distortion using an ultrasonic multi-beam sensor.

When matters such as gas and air go through the ultrasonic sensor of an existing sludge density meter, the measured value can be overstated or can fluctuate like a flow measurement [7,8]. When a sludge feeding pump is operated, initially the sludge suddenly moves in a congested state, thus, time is needed for it to stabilize and to get an exact value. Widely used insertion-type density meters might deteriorate the sensor sensitivity due to sludge attachment on the sensor surface, which could cause more errors. To overcome these problems, an ultrasonic multi-beam concentration sensor was considered. An abnormal concentration value of a specific ultrasonic beam, however, degrades the accuracy of the entire measurement in case of using a conventional arithmetic mean for all measurement values. Figure 1 shows that Sensor-3 is disturbed by bubbling inside the pipe among four sensors and as a result, the arithmetically averaged measurement value gives a rather larger measurement error.

In this paper, we propose a method to improve the accuracy of the sludge concentration determination by choosing reliable sensor values and applying a neuro-fuzzy learning algorithm. The newly developed meter is proven to render useful results from a variety of experiments performed on a real water treatment plant.

## 2. Development of the Ultrasonic Multi-Beam Densitometer

### 2.1. Development Overview

A detachable sensor instrument needs to be considered for easy cleaning, calibration and maintenance in addition to real-time sludge concentration measurement in sludge treatment processes in water purification plants (adjustment ↔ thickener ↔ storage tank ↔ dehydrator) and sewage treatment plants (primary sedimentation, thickener, aeration, secondary sedimentation basin, storage tank, dehydrator, *etc*.). The effects of air bubbles were minimized by filtering the measured values of the multi-beam method, which kept the output value stable.

### 2.2. Densitometer Configuration

**Figure 2:**
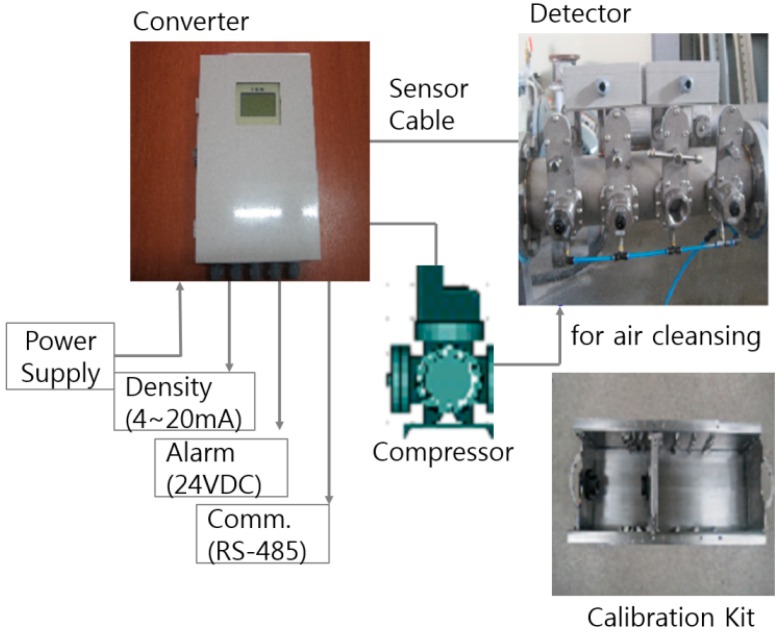
Structure of the ultrasonic concentration meter.

In Figure 2, the diagram shows the structure of the developed ultrasonic densitometer which consists of a flange to be attached to the pipe, a sensor to measure its attenuation, a converter to be changed to an electric signal and a display to show its measured value. In addition, a compressor and portable calibration kit are built inside for periodic air cleansing and accuracy improvement.

### 2.3. Converter Design and Function

**Figure 3:**
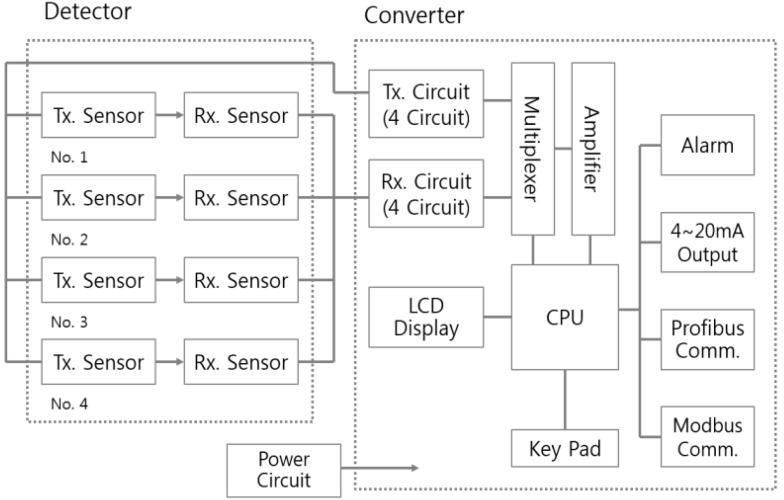
Block diagram of the convertor circuit.

Table 1 shows the design specification of the converter, which is considered to minimize the influence of gas and bubbles which are known to cause lower or abnormal measurements. As the block diagram of the meter in Figure 3 shows, four ultrasonic sensors measure the values, calculate a mutual deviation and then remove the largest abnormality which is likely to be calibrated poorly and influenced by air.

**Table 1:** Converter design specifications.

Item	Design Specifications
**Ultrasonic Circuit**	Calculates the attenuation value using the measured voltage of the receiving circuit in case of the 3 MHz AC signal transmission
**Display**	LCD WG1286A 128X64DOT
**Converter**	Switches the signal of the transmitter and reception circuit through the amplifier circuit. Multiplexer MX399
**Amplifier**	Amplifies the outgoing signal so that the received signal is at a constant level, Amplifier VCA810 GAIN 80 dB (±40 dB)
**CPU**	CPU HD64F2357F (Hitachi, 16BIT Micro Processor)
**Memory**	ROM 128 kByte, RAM 8 kByte, External Memory(RAM 256 kByte), Clock 20 MHz
**Alarm**	Dry Contact and High/Low of Density
**Output**	4–20 mA, RS485 (MODBUS) Comm.

**Figure 4:**
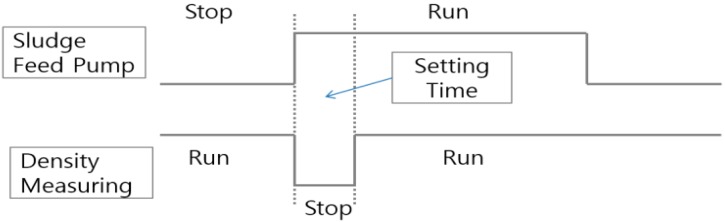
Delay of the density measurement.

If the sludge transfer pumps are started, initially submerged sludge already in pipeline gives a non-uniform shape, which causes measurement errors. To solve this problem, as shown in Figure 4, the timing of the density measurement is delayed for a certain period of time after the initial start-up of the sludge feed pump operation (delay time: up to 300 s).

### 2.4. Design and Function of the Detector

**Figure 5:**
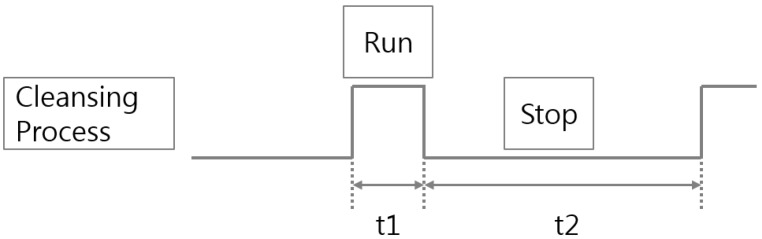
Flow chart for the auto clarifier operation.

A newly developed stainless steel detector is designed in order to prevent the corrosion and suppress sludge deposition by curving the surface of the sensor and using a directional ultrasonic wave. The ultrasonic sensor cable is designed as a connector type for convenience of detaching and attaching the ultrasonic sensor. The water resistance level is chosen as IP 67 for easy portable calibration. The Tx and Rx sensors are designed to accommodate multiple sensors, in this paper, a set of four. In addition, a compressor was built into the panel in order to suppress the deposition of sludge on the sensor during operation, as shown in Figure 5. Air is periodically injected into the sensor surface through the holes (1.5 mm) of the lower end of sensor to prevent interference of the fluid flow, so it is called self-cleaning.

A converter is designed to allow the removal of sensors without interrupting the sludge treatment processes, which makes the present bypass pipeline and valve useless and saves space and cost. The sensors can be easily removed and attached by local operators, which usually must be done by professional engineers and with tools.

### 2.5. Design and Function of the Calibration Kit

A portable calibration device can be applied to a variety of pipe diameters can complete calibration work quickly and easily within 2 h. Ultrasonic sensors are inserted into both sides of the calibrator, where the ultrasonic transmitting plate is fixed inside and the receiving plate is flexible so the position for each diameter can be adjusted. At first, the sensor calibrator is inserted in the receiving plate with the same portable sensor diameter as the ultrasonic sensor. Then, transmitting and receiving sensors are removed from the calibrated density meter and attached into a portable calibration device. Zero calibration is completed by entering the ultrasonic attenuation measurement by ultrasonic sensor channels in the clear water. Laboratory calibration is also done by entering the attenuation value after injecting the laboratory reagent, kaolin. Calibration processes should be carried out individually and repeatedly, because the attenuation between each channel is different.

## 3. Sludge Density Estimation Algorithm Using Neuro-Fuzzy Method

**Figure 6:**
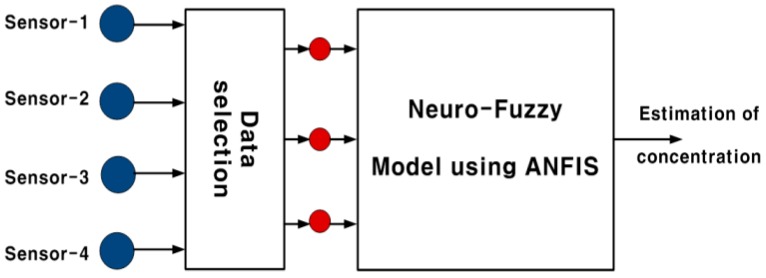
Concentration estimation algorithm using a neuro-fuzzy model.

The proposed algorithm uses a data selector to choose data from three of the sensors with lower disturbance among the four sensors with input selection, as shown in Figure 6, and then sludge density is estimated through a neuro-fuzzy algorithm for the three selected sensor data.

### 3.1. Data Selection

**Figure 7:**
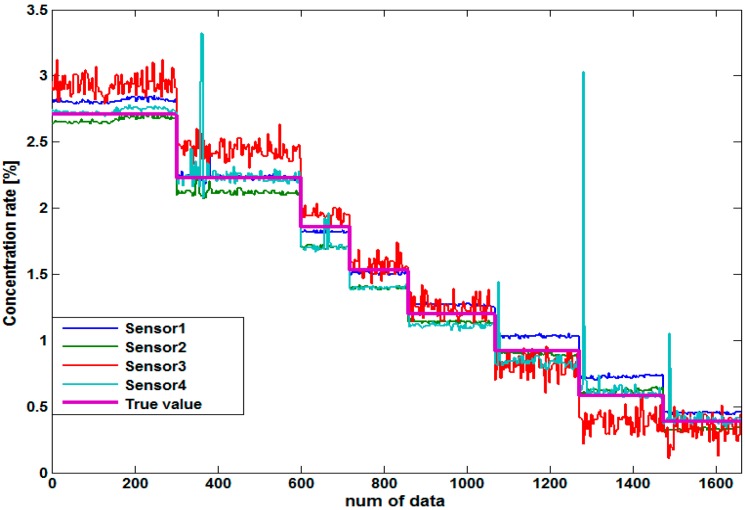
Concentration value acquired by four sensors.

Data preprocessing is done to eliminate the largest deviating data. That is, the Euclidean distances between the values of four sensors are calculated and then the sensor having the largest deviation is removed in every data point, which is thought to be due to disturbance by bubbles or lumps of sludge. For a kaolin solution test in a pilot plant, Sensor 1 and Sensor 2 are relatively stable as shown in Figure 7. Sensor 3, however, has large amplitudes that change continuously. On the other hand, Sensor 4 is closest to the actual measured value, but strange changes suddenly occur three times due to disturbance factors.

**Figure 8:**
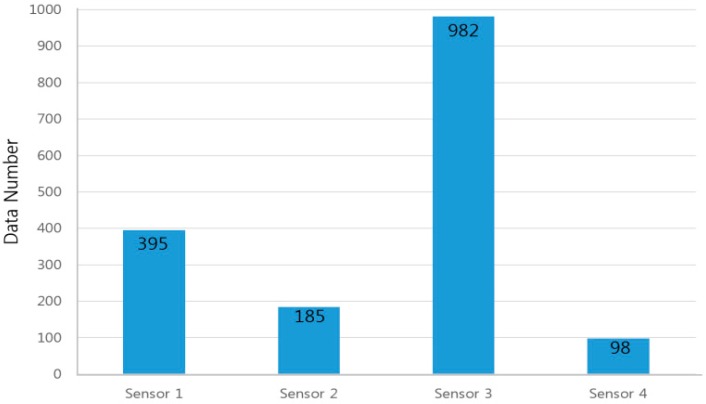
Frequency of sensor removal by the data selector.

In this case, Figure 8 shows the results of input selection removal data of sensors, where Sensor 3 has been removed most frequently at 982 times, while Sensors 1, 2, and 4 were removed 395, 185, and 98 times, respectively. It is shown that only one dominant sensor does not continuously and significantly affect the measurement error. Since bubbles and the mass of sludge are moving in the pipe, they can affect any sensor among the four sensors. So, if the values of Sensor 2 or Sensor 4 deviate from the other sensor values, they can also be removed.

### 3.2. Neuro Fuzzy Algorithm

**Figure 9:**
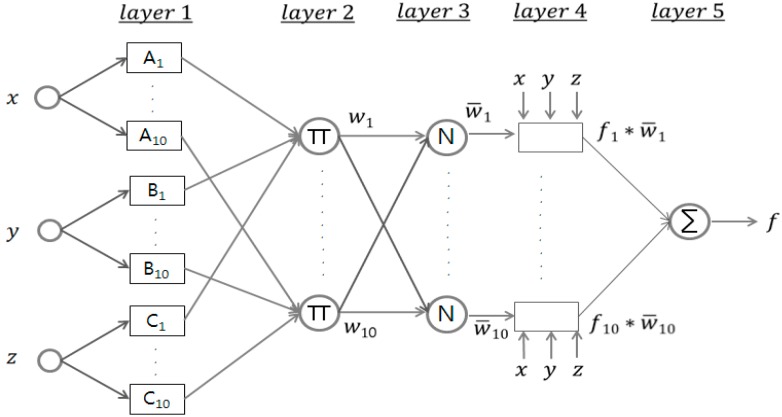
ANFIS structure for density inference.

Figure 9 shows the neuro-fuzzy algorithm structure for the sludge concentration forecasting. For the concentration predictions calculated by the proposed algorithm, each learning was based on three sensors after removing the sensor having the largest deviating value.

The Adaptive Network-based Fuzzy Inference System (ANFIS) uses the Tagagi-Sugeno-Kang (TSK) fuzzy structure, which is believed to be computationally efficient and amendable to elegant mathematical analysis. The characteristics and learning processes of ANFIS with Matlab Fuzzy Logic Toolbox are as follows [9,10,11,12].

Layer 1: Let us denote x, y and z as selected three sensor outputs, then every node i in this layer, calculate the membership value for the premise parameter as shown in Equation (1):

(1) $$\begin{array}{c} \;O_{i}^{1}=\;u_{A_{i}}\left(x\right)\;for\;i=1\sim 10,\\ O_{i}^{1}=u_{B_{i-10}}\left(y\right)\;for\;i=11\sim 20\\ \;\;O_{i}^{1}=u_{C_{i-20}}\left(z\right)\;for\;i=21\sim 30 \end{array}$$

Here, a generalized bell function is selected as membership function for the fuzzy set $\;D\;(=A_{i},\;B_{i}\;or\;C_{i}$) as shown in Equation (2):

(2) $$u_{D}\left(x\right)=\frac{1}{1+{\left|\frac{x-c_{i}}{a_{i}}\right|}^{2b_{i}}}\;$$

where, $\left\{a_{i},b_{i}\;,c_{i}\right\}$ is the parameter set.

Layer 2: Output the weight to multiply the membership value obtained in layer 1 by each rule:

(3) $$O_{i}^{2}=w_{i}=\;u_{A_{i}}\left(x\right)\times u_{B_{i}}\left(y\right)\times u_{C_{i}}\left(z\right),\;\;\;i=1,\;2,\;\cdots ,\;10\;$$

Layer 3: Calculate ratio of i-th rule’s strength vs. all rules’ firing strength and normalize firing strength as shown in Equation (4):

(4) $${\text{O}}_{\text{i}}^{3}=\bar{{\text{w}}_{\text{i}}}=\frac{{\text{w}}_{\text{i}}}{{\text{w}}_{1}+{\text{w}}_{2}+\cdots +{\text{w}}_{10}},\text{ i}=1,\;2,\;\cdots ,\;10$$

Layer 4: Calculate the multiplication of normalized value and consequent parameters for each node as shown in Equation (5):

(5) $$O_{i}^{4}=\bar{w_{i}}f_{i}=\bar{w_{i}}\left(p_{i}x+q_{i}y+r_{i}\text{z}+d_{i}\right),\;i=1,\;2,\;\cdots ,\;10$$

where, $\bar{w_{i}}\;$ are normalized firing strengths obtained in Layer 3 and $p_{i},\;\;q_{i},r_{i}\text{ and }d_{i}$ are the linear parameters of Tagaki-Sugeno fuzzy modeling [13].

Layer 5: Calculate overall output by using weighted average:

(6) $$O_{i}^{5}=\overset{10}{\underset{i=1}{\sum }}\bar{w_{i}}f_{i}=\frac{\sum^{\text{​}}w_{i}f_{i}}{\sum^{\text{​}}w_{i}}$$

**Figure 10:**
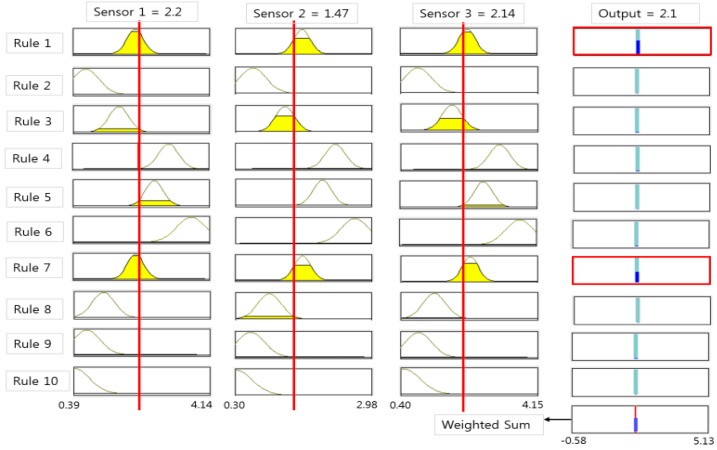
Fuzzy rule and inference value for density estimation.

Figure 10 shows a neuro-fuzzy learning process to be completed for the sludge concentration prediction and to be composed of 10 fuzzy clusters and rules. Each fuzzy membership of the selected three sensor inputs is calculated and minimum membership values are taken in each rule. Then, a final output is calculated by the weighted sum of the output and its weight [14,15].

## 4. Experimental Results

### 4.1. Configuration of the Experimental Device

**Figure 11:**
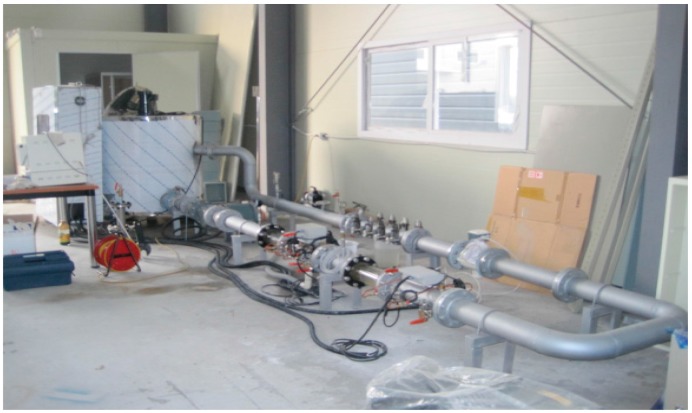
Pilot plant system.

A pilot plant is configured as shown in Table 2 and Figure 11 for the performance test of the multi-beam sludge concentration meter. Its main components are composed of a sludge storage tank, stirrer for mixing, inverter pump for the circulation of the mixed sludge, electromagnetic flowmeter, multi-beam ultrasonic meter, and data logger.

**Table 2:** Major facilities of the pilot plant.

Type	Specifications	Remarks
Sludge Tank	5 m^3^	Capacity
Mixer	0.75 kW	
Supply Pump	Inverter 2.2 kW	Sample Circulation
Magnetic Flowmeter	100 A	Velocity measurement
Ultrasonic Density meter	4 CH 100 A	
RTU	Cimon	Real Time data logging
Electronic Scale	200 g	ACCULAB
Oven	550 °C	Lab House

The ultrasonic meter is designed and manufactured in order to mount a set of four multiple beam sensors. The detector is made with stainless steel to prevent corrosion, and the sensor surface was curved as much as possible for the improvement of the ultrasonic directional strength, which also helps to suppress the deposition of sludge. The ultrasonic sensor cable is manufactured as a connector type for convenient attachment and detachment, and a IP67 rating is taken into account for usage as a portable calibration device.

### 4.2. Experimental Method

Fresh water is mixed with kaolin powder similar to sludge characteristics, by which various concentrations of sludge are created in order to be tested in the pilot plant. Before data is acquired from the densitometer sensor, the sensor needs zero and span calibration because it depends on the characteristics of installation conditions and manufacture status.

**Figure 12:**
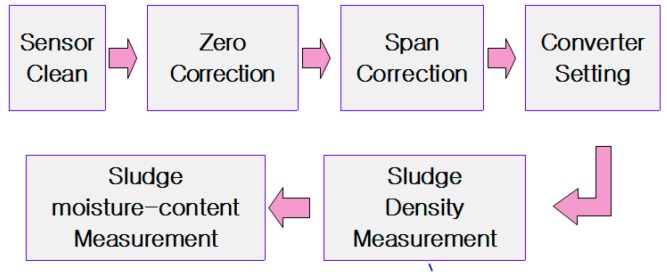
Experimental procedure for the pilot plant.

The pilot plant test procedures are conducted as shown in Figure 12. Data are acquired in real-time by the second through a Human Machine Interface (HMI) software installed on a laptop computer, which is connected with the densitometer converter through RS-232 communication.

The sensor clean step removes foreign substances on its surface in order to reduce the measurement error before calibration. In the zero calibration step, the reception attenuation displayed on ultrasonic sensor transducer is set to a concentration value of 0%, in which fresh water is filled in the densitometer. The span setting is calibrated by comparing a 3% concentration of kaolin solution with the measurement value for the attenuation of the ultrasonic sensor transducer. The converter setting involves the connection and acquisition of data through an RS-232 interface with a laptop computer in the densitometer converter. Sludge density measurement involves the measurement of the real-time concentration for the sludge supplied by a feed pump from a tank.

Finally, the sludge moisture-content measurement step involves measuring the water content of sludge, in which the weight of the evaporation dish ($g_{1}$) and the weight of a sample liquid ($g_{2}$) in an evaporating dish are measured. Then the weight ($g_{3}$) is measured again after drying by heating for 12 h or more in an oven at 105 °C. The water content is calculated as the ratio of the solid ($g_{3}$) and the sludge ($g_{2}$). The concentration calculation formula is shown below:

(7) $$\text{Density}\left(\text{\%}\right)=\frac{\left(g_{3}-g_{1}\right)}{\left(g_{2}-g_{1}\right)}\times 100\;$$

A scale and oven are used to know the true sludge density, where the scale is a model ALC2100.2 produced by ACCULAB (Tokyo, Japan) and the oven is a model DMF-5T produced by LAB house (Pocheon-si, Gyeoggi-do, Korea).

### 4.3. Experimental Results

Various algorithms are reviewed in order to improve the measuring accuracy of the meter from the acquired data. General methods and intelligent algorithms are compared with each other to improve the measurement accuracy of the sludge concentration meter. The algorithms are verified through sample kaolin solution and sludge in a water treatment plant in order to evaluate the performance measurement. In addition, outlier removal algorithms are adopted to minimize the error according to mass and air bubbles. The algorithms are shown in Table 3 to review the sludge concentration prediction.

**Table 3:** Tested algorithms for sludge density estimation.

Method	Algorithm
Method 1	Arithmetic mean with four sensors
Method 2	Arithmetic mean with selected three sensors
Method 3	Multiple regression with four sensors
Method 4	Multiple regression with selected three sensors
Method 5	Neuro fuzzy with four sensors

Mean Absolute Percentage Error (MAPE), Mean Absolute Error (MAE), Root Means Square Error (RMSE) and R-squared are considered for the algorithm performance evaluation:

(8) $$\text{MAPE}=\frac{1}{N}\overset{N}{\underset{i=1}{\sum }}\left|\frac{Actual_{i}-Forecast_{i}}{Actual_{i}}\right|\times 100$$

(9) $$\text{MAE}=\frac{1}{N}\overset{N}{\underset{i=1}{\sum }}\left|Actual_{i}-Forecast_{i}\right|$$

(10) $$\text{RMSE}=\sqrt{\frac{1}{N}\overset{N}{\underset{i=1}{\sum }}{\left(Actual_{i}-Forecast_{i}\right)}^{2}}$$

#### 4.3.1. Kaolin Solution Test in the Pilot Plant

We use a kaolin solution which can easily change its concentration. It is measured by multi-beam sensors only to estimate its density by the proposed algorithms. The data set composed of desired input-output pairs is called the training data and the data set to see if the model identified responds correctly is referred to as the test data [13]. A total of 2920 datapoints are measured and averaged in arithmetic mean method without the division of training and testing data. However, even-indexed and odd-indexed data are divided into training and testing data equally for the linear regression and neuro- fuzzy models to check the over-fitting and under-fitting error.

**Table 4:** Experiment result by kaolin solution in the pilot plant.

**Algorithm**	**All Data**			
	**MAPE**	**MAE**	**RMSE**	**R-Squared**
One Sensor	28.8819	0.6554	0.7212	0.1164
Method 1	13.5682	0.2603	0.2781	0.9239
Method 2	8.9533	0.1459	0.1604	0.9783
**Algorithm**	**Training Data**			
	**MAPE**	**MAE**	**RMSE**	**R-Squared**
Method 3	3.3183	0.0638	0.0820	0.9940
Method 4	5.2251	0.0817	0.1073	0.9903
Method 5	0.0431	0.0003	0.0011	1.0000
Method 6	0.0009	0.0001	0.0001	1.0000
**Algorithm**	**Testing Data**			
	**MAPE**	**MAE**	**RMSE**	**R-Squared**
Method 3	3.3123	0.0637	0.0820	0.9941
Method 4	5.2335	0.0815	0.1072	0.9904
Method 5	0.0424	0.0003	0.0011	1.0000
Method 6	0.0009	0.0001	0.0001	1.0000

Table 4 shows the analysis results by the arithmetic means and the learning algorithms with and without outlier removal. The percentage error of one sensor is 28%, which can vary by sensor, before multiple sensors are used. Multiple sensors can improve the error to 13% using the conventional arithmetic mean. If the outlier removal method is added to remove the data with the largest deviation among four sensors, arithmetic mean without outlier can reduce its error to 8.9%. The conventional linear regression method improves the error to 3% and the proposed algorithm (neuro fuzzy with outlier removal) has the lowest error of 0.0009%.

**Figure 13:**
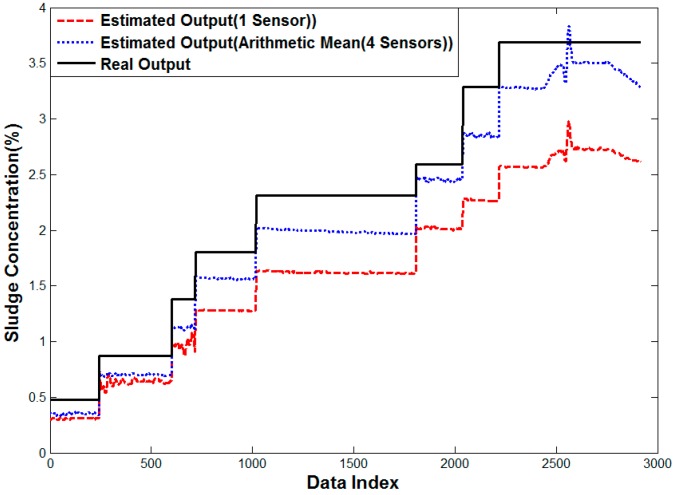
Performance curve of one sensor case and Method 1.

Figure 13 shows the analysis results of the error characteristics for one sensor. As the sludge concentration is increased, the measurement error also increases. It is assumed that 2500th data values have a sudden high error due to bubbles or mass of sludge. The error characteristic for arithmetic mean using four sensors is improved when compared with one sensor in a high concentration. Most of estimated values are lower than the actual measured value. It is thought that the difference is caused by the off-set depending on the fluid status such as bubble and sludge attachment to sensors.

**Figure 14:**
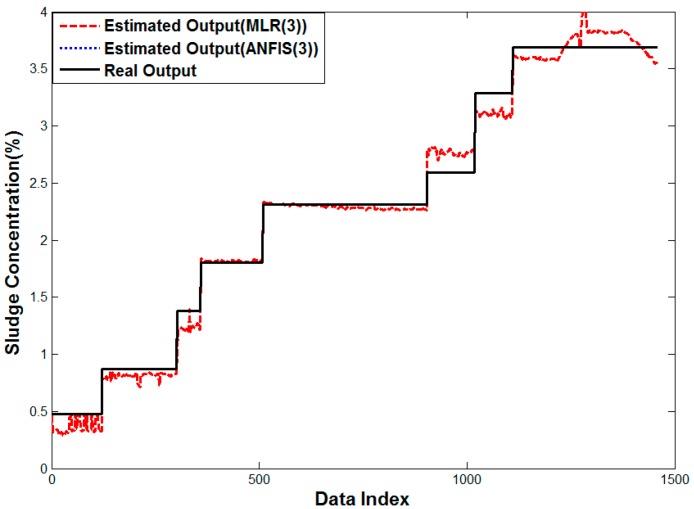
Performance curve of Methods 4 and 6.

Figure 14 is the result of a linear regression analysis and ANFIS with input selection, which helps minimize the estimation error. The estimated values are located around the true value contrary to arithmetic mean. While the error of linear regression still exists a little more in high density areas, the error of ANFIS cannot be noticed because the true and estimated values are overlapped. In the error analysis with histograms, it ranges from −0.5 × 10^−4^ to 0.5 × 10^−4^.

#### 4.3.2. Real Sludge Test in the Pilot Plant

Real sludge was gathered from the sludge thickener at an operating water treatment plant, retained for two days and a high concentration sludge from a lower layer was extracted. The test was done with the highly concentrated sludge diluted with water.

A total of 1600 datapoints are averaged in the arithmetic mean method without input selection. In the linear regression and neuro- fuzzy models, all data is equally divided into training and testing data. Table 5 shows the test results of the water treatment plant sludge in the pilot plant, in which one sensor has an error of 12.6% while the multi-sensor has an error of 3%–4%. As it is known in the linear regression analysis, more variables result in better performance. The proposed neuro-fuzzy algorithm with three sensors subjected to input selection, however, is the lowest with an error of 0.01%.

**Table 5:** Experiment result by WTP sludge (pilot).

**Algorithm**	**All Data**			
	**MAPE**	**MAE**	**RMSE**	**R-Squared**
One Sensor	12.6388	0.1459	0.1722	0.9677
Method 1	3.1092	0.0412	0.0573	0.9954
Method 2	4.0933	0.0453	0.0694	0.9926
**Algorithm**	**Training Data**			
	**MAPE**	**MAE**	**RMSE**	**R-Squared**
Method 3	2.5247	0.0305	0.0385	0.9978
Method 4	5.4544	0.0560	0.0632	0.9941
Method 5	0.0271	0.0002	0.0004	1.0000
Method 6	0.0172	0.0001	0.0003	1.0000
**Algorithm**	**Testing Data**			
	**MAPE**	**MAE**	**RMSE**	**R-Squared**
Method 3	2.5358	0.0310	0.0388	0.9977
Method 4	5.4620	0.0553	0.0625	0.9942	
Method 5	0.0276	0.0002	0.0004	1.0000
Method 6	0.0159	0.0001	0.0003	1.0000

**Figure 15:**
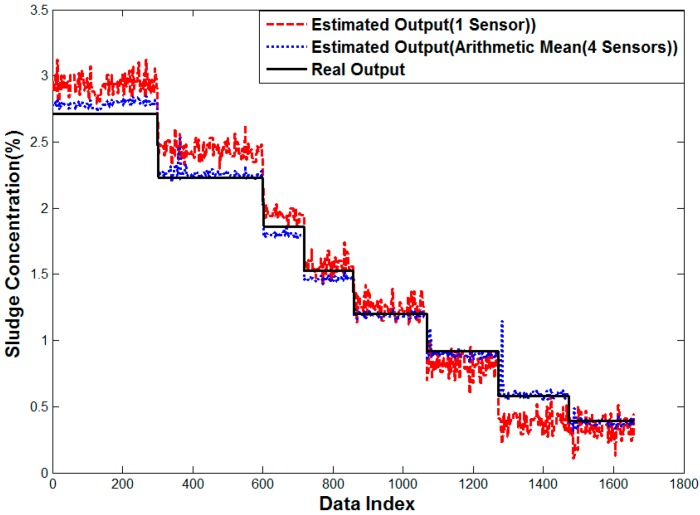
Performance curve of the one sensor case and Method 1.

Figure 15 shows the error analysis of one sensor, where the estimated value is higher in terms of high density but it is lower in terms of low density. In the error analysis of four sensors, the estimated value is especially lower in the middle density and the other area has a sudden peak of data several times compared to one sensor case. It is assumed to be caused by bubbles and a mass of sludge.

**Figure 16:**
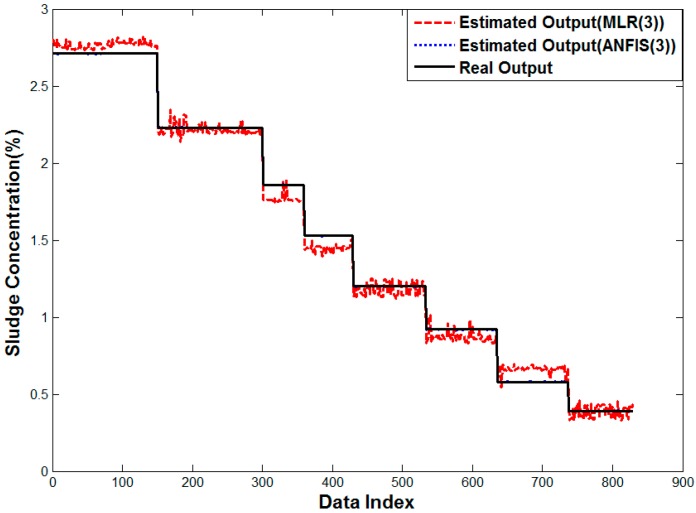
Performance curve of Methods 4 and 6.

Figure 16 shows the result of multiple linear regression analysis, where the error in the arithmetic mean is smoothed, but the rather small error is observed in all densities. The outlier shown in Figure 15 is removed, because the sensor affected by the distortion would have a lower weight. On the contrary, the proposed neuro-fuzzy algorithm with input selection gives almost the same result as the real value. Therefore, the proposed method renders the best solution to estimate the density for real sludge tests in the pilot plant.

#### 4.3.3. Real Sludge Test in a Real Water Treatment Plant

The developed multi-beam density meter was installed and operated in a real water treatment plant in Korea. The data obtained from the meter was compared with the data from an actual water analysis method. The total data is a 4990 data set, which are equally divided into learning and testing data. Table 6 shows the results of tests in the actual operating water treatment plant. When using only one sensor, the error was 2.74% and whereas the multi-sensor has a 0.1 to 0.6 percent error. The multi-linear regression method has 0.1% error and neuro fuzzy algorithm has the least error at 0.02%.

**Figure 17:**
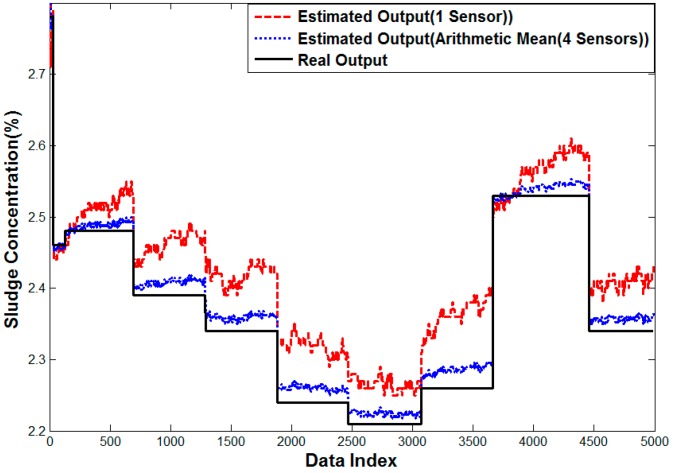
Performance curve of one sensor case and Method 1.

Figure 17 shows the results of analysis of the error characteristics using only one sensor and the arithmetic mean on the four sensors. While one sensor case has the error between −0.05 to 0.15, which is a little biased towards the positive value to be corrected, The error of four sensor average method is greatly improved compared with the one sensor and is close to the true value in all sectors.

**Table 6:** Experiment result by WTP Sludge (WTP).

**Algorithm**	**All Data**			
	**MAPE**	**MAE**	**RMSE**	**R-Squared**
One Sensor	2.7421	0.0638	0.0696	0.5204
Method 1	0.6932	0.0161	0.0176	0.9748
Method 2	0.0962	0.0023	0.0027	0.9994
**Algorithm**	**Training Data**			
	**MAPE**	**MAE**	**RMSE**	**R-Squared**
Method 3	0.0984	0.0023	0.0027	0.9994
Method 4	0.4288	0.0100	0.0138	0.9852
Method 5	0.0221	0.0006	0.0012	0.9999
Method 6	0.0191	0.0005	0.0012	0.9999
**Algorithm**	**Testing Data**			
	**MAPE**	**MAE**	**RMSE**	**R-Squared**
Method 3	0.0974	0.0023	0.0027	0.9994
Method 4	0.4240	0.0099	0.0137	0.9856
Method 5	0.0224	0.0006	0.0012	0.9999
Method 6	0.0190	0.0005	0.0011	0.9999

**Figure 18:**
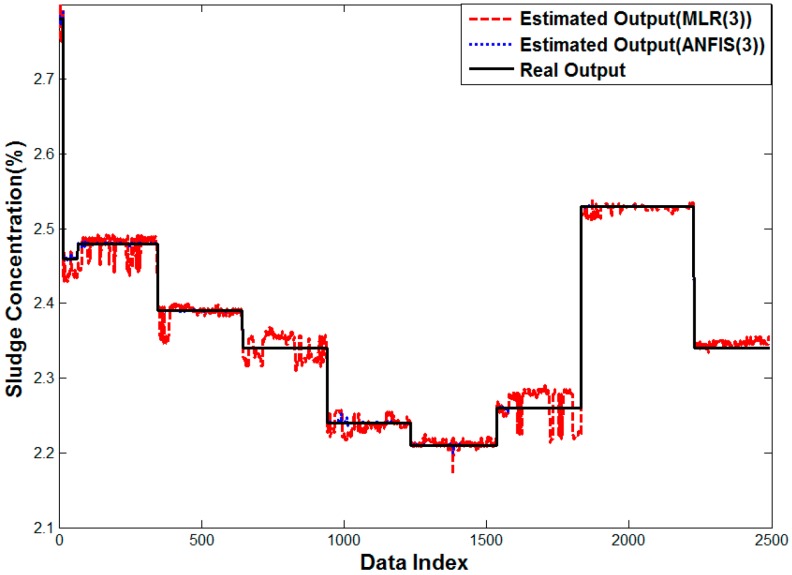
Performance curve of Methods 4 and 6.

Figure 18 shows the result of multiple linear regression analysis and ANFIS for three sensors. The error in arithmetic mean almost disappears, because it helps to overcome the bias in the conventional average method by analyzing the past data. The proposed neuro-fuzzy system with input selection has the least error as shown in Figure 18. Thus, the proposed method is shown to estimate the sludge density properly also in case of the real plant.

## 5. Conclusions

Sludge concentration meters, which are mostly calibrated at initial installation to achieve the automation, are very important to automate sludge treatment processes and polymer injection. However, they are likely to contain bubbles or debris because of the sludge characteristics and fouling, and sensors can be easily distorted in specific density range. In addition, sludge is deposited on the sensor unlike in fresh water, which causes errors and failures. Thus, the multi-beam densitometer is developed and new algorithms are proposed to solve these problems and improve its reliability.

This paper proposes a method to improve the accuracy of sludge concentration determinations by choosing reliable sensor values and learning them by a neuro fuzzy algorithm, which is tested by the various experiments in a pilot plant and a real water treatment plant. It is shown that estimation of sludge density requires more sensors to increase the number of accurate measurements and the outliers needs to be sorted by an input selection algorithm to find the sensor with the largest deviation. Even though the pre-processing results show inconsistency in the linear method, the proposed neuro-fuzzy algorithm with multiple sensors and the input selection is proven to improve the performance significantly both in the pilot plant and a real water treatment plant.
